# Effects of Weightlessness on Molecular Changes in Cellular Organisms, Animals and Plants

**DOI:** 10.3390/biom15081207

**Published:** 2025-08-21

**Authors:** Daniela Grimm

**Affiliations:** 1 Department of Microgravity and Translational Regenerative Medicine, Otto-von-Guericke-University Magdeburg, 39106 Magdeburg, Germany; daniela.grimm@med.ovgu.de; 2 Research Group “Magdeburger Arbeitsgemeinschaft für Forschung unter Raumfahrt- und Schwerelosigkeitsbedingungen” (MARS), Otto-von-Guericke-University Magdeburg, 39106 Magdeburg, Germany; 3 Department of Biomedicine, Aarhus University, 8000 Aarhus, Denmark

## 1. Introduction

Space travel is a dream of humankind. Planned deep space exploration adventures will increase the time which humans or animals will spend in weightlessness. Space travelers will be exposed to microgravity (µ*g*), cosmic radiation, isolation and other stresses. Unfortunately, a long-term spaceflight has large well-known effects on our health [1]. To counteract these health problems, studies supported by NASA, ESA and other space agencies focusing on cardiovascular changes, bone loss, muscle atrophy or the immune system, among others, have been performed in recent years [2,3]. Space research is a hot topic at present. To perform research in weightlessness, several platforms with diverse durations of µ*g* exposure for experiments in real µ*g* are available [4]. A drop tower provides about 4.74 s of weightlessness up to three times a day. The time in µ*g* can be doubled with a catapult system. With the catapult, the experimental subjects are flung from the bottom to the top and then fall back. A drop tower is mainly used for experiments in science and physics under space conditions [5,6]. Short-term experiments in µ*g* can be realized during a parabolic flight [7]. A parabola provides 22 s of µ*g* and two phases of hypergravity lasting 20 s. Researchers can study the early responses to gravitational changes in humans, microorganisms, cells and plants, among others. Parabolic flights are used for astronauts’ training and human physiological experiments, as well as in physics and material sciences. Other platforms, like sounding rockets, can provide, depending on the rocket type, from 3 min up to 15 min of µ*g*. These rockets carry scientific instruments and experiments into space along a parabolic trajectory [8]. Sounding rocket flights are used for short-term studies in medical, biological, physical, chemical, atmospheric or material sciences [8,9,10]. To perform long-term studies (days), biosatellites like the Russian Bion spacecraft or the Shenzhou spaceships can be used. The International Space Station (ISS) and the Chinese Space Station (CSS) provide the possibility to perform experiments lasting more than six months [11,12].

Unfortunately, experiments in real µ*g* on the ISS and CSS are expensive and rare. It is difficult to obtain the opportunity to perform studies in space. Therefore, so-called ground-based facilities (GBFs) to simulate µ*g* have been developed. NASA and ESA have acknowledged various GBFs. Examples include the NASA-developed rotating wall vessel (RWV) and the 2D and 3D clinostat, among others such as the Random Positioning Machine (RPM). All devices are able to create µ*g* on Earth. Researchers use these machines to prepare space missions, or to investigate the cell- and molecular-level biological effects of µ*g* on cells, microorganisms, bacteria or plants [13]. Frequent research objectives in the field of space biology are to study changes in protein and gene expression or the cell metabolism of cells, animals and humans, or plants exposed to µ*g*. Omics investigations in weightlessness focusing on the human and animal organism or on cells aim to characterize and quantify the biological factors responsible for the observed changes in morphology, structure, function, metabolism and dynamics of cells exposed to µ*g* conditions. Weightlessness has been demonstrated to influence various biological processes in vitro and in vivo. Increasing our knowledge in this research field could help us find countermeasures against various space stressors and may improve our understanding of various diseases on Earth.

## 2. An Overview of Published Articles

This Special Issue (SI), “***Effects of Weightlessness on Molecular Changes in Cellular Organisms, Animals and Plants***”, comprises research articles in the field of gravitational biology, space medicine, translational regenerative medicine and molecular oncology. This issue contains nine research articles [14,15,16,17,18,19,20,21,22] and one review [23] (Table 1). It addresses the impact of µ*g* on benign human [14,17] and animal cells [22], stem cells [15,18], cancer cells [21], freshwater protists (*Euglena (E.) gracilis*) [19], mice [16] and rats [20].

It has long been known that mammalian cells change their growth behavior, differentiation, proliferation, adhesion, etc., when cultured under µ*g* conditions in space and on Earth [24,25]. This SI presents three publications supporting these findings and demonstrating the three-dimensional (3D) growth of different cell types exposed to s-µ*g* [14,17,21]. Jokšienè et al. [14] studied endothelial cells under s-µ*g* using a 3D clinostat for 14 d in high-glucose (HG) and normal-glucose (NG) media. The authors found changes in growth. One portion of the endothelial cells grew in the form of 3D aggregates (spheroids), the other as a monolayer. The HG medium induced bigger and more spheroid growth compared to NG medium. An earlier study showed that 3D tissue-like aggregates can be engineered on the RWV [26]. In addition, angiogenic activity was also observed when adipose-derived mesenchymal stem cells were exposed to an RPM [27].

Tissue engineering in µ*g* is currently a hot topic [28]. Culturing human cells like chondrocytes, under µ*g* conditions, are suitable for the tissue engineering of cartilage [29]. Steinwerth et al. [17] demonstrated that the RWV is suitable for engineering cartilage tissues without any scaffolds. At present, experiments in space and studies using µ*g*-simulating devices are increasingly performed in the field of cancer research [30,31,32,33].

The lessons learnt from cancer research in space, among other things, showed that cancer cells exposed to µ*g* reveal changes in growth behavior. The µ*g* environment induces multicellular spheroids and organoids, with the potential to study the processes of cancer progression and metastasis on a molecular level. Barkia et al. [21] investigated two types of lung cancer cells on the RPM and demonstrated that NCI-H1703 squamous cell carcinoma cells and Calu-3 adenocarcinoma cells exhibit a different growth behavior after 3 d. A 3-day RPM exposure induced stable spheroid formation in NCI-H1703 cells but not in Calu-3 cells. Mucin-1 was reduced in Calu-3 cells and further decreased under µ*g* conditions. RPM-exposed Calu-3 demonstrated sensitivity to 17β-estradiol or phenol red, allowing for the formation of more stable spheroids.

Moreover, this SI covers three publications reporting on studies performed on the ISS [15,16,18]. One group reported two different stem cell experiments [15,18]; another one reported the results of an animal study [16]. The group of Dr. Espinosa-Jeffrey investigated oligodendrocyte progenitors on the ISS and found that the proliferation and autophagic death of the cells were enhanced after spaceflight [15]. The secretome of space-flown cells revealed that HSP-90 and SPARC proteins were clearly elevated, indicating their key role in the mitigation of intracranial hypertension and the visual impairment intracranial pressure of space travelers during and after a space mission [15]. In a second ISS experiment, neural stem cells were studied. The authors demonstrated that spaceflight enhances stress pathways in human neural stem cells [18]. Interestingly, they found an increased secretion of the ER stress-related proteins SPARC, calreticulin and endoplasmin following spaceflight and the induction of autophagy-like behavior in neural stem cells was detected after spaceflight [18]. The third ISS experiment is an animal study [16]. The authors focused on the transcriptomic changes in the hearts of female C57BL/6J mice flown on the ISS for 30 d. Only 1147 transcripts (1.3%) were significantly regulated in the flight group, suggesting that by 30 d the heart successfully adapted to the stressful space conditions. Activated biological processes in the flight group contributing to adaptation and survival included the PI3K-Akt pathway, GPCR signaling pathway and MAPK signaling pathway. Transcriptomic analyses predicted the activation of these pathways. The authors concluded that the heart adapts to µ*g* during and after long-term spaceflight [16].

A second animal study investigated rats under s-µ*g* conditions [20]. For this purpose, the authors used the standard rodent tail suspension model and focused on liver tissue. Proteomics and metabolomics results revealed lipid metabolism disorder in the rat liver [20]. S-µ*g* induced abnormal changes in proteins and metabolites involved in the PPAR signaling pathway. These changes may be the primary cause of lipid metabolism disorders in rat livers [20].

A further study examined porcine epithelial cells (IPEC-1 and IPEC-J2) under dynamic mechanical load [22]. The results demonstrate the impact of motion on the intestinal epithelial border cells in vitro. The cultivation conditions obtained by a rotating vessel within 3 d were sufficient to trigger significant morphological and cell physiological modifications (growth and proliferation), which improved the expressiveness of the IPEC cell culture model [22].

Krüger et al. examined the transcription levels of four independent *E. gracilis* cultures [19]. The gene expression and phenotype of *E. gracilis* cells change depending on age. mRNAs changes were measured in older cells. DEGs were detected for adenylyl cyclases, photosynthesis and metabolic enzymes. Regarding the significance of *E. gracilis* for biotechnological, pharmacological and space applications like biological life-support systems, the determination of such shifts is very important [19].

Finally, Farva and coauthors reviewed the current knowledge regarding the effects of real and simulated µ*g* on the metabolism and signaling of bioactive lipids in the context of immune and inflammatory homeostasis [23].

## 3. Conclusions

The excellent papers included in this SI report novel findings in the field of space research and space medicine. Space stressors like µ*g*, radiation, isolation and psychological problems are known to impact the health of humans and animals traveling to space. Therefore, it is necessary to study the mechanisms of these health problems and to find suitable countermeasures [34,35].

This SI covers new publications focusing on omics studies of cells and tissues exposed to conditions of real and simulated weightlessness, with a focus on the transcriptome, proteome, secretome and metabolome. In addition, new data regarding the tissue engineering of cartilage, vessels and lung cancer spheroids have been published. Dynamic mechanical load serves as a trigger for growth and proliferation in porcine epithelial cells. Furthermore, novel ISS results with respect to the stressors for intracranial hypertension and visual impairment intracranial pressure, as well as adaptation of the mice heart to long-term spaceflight, are provided. Data regarding lipid metabolism disorders in the liver of rats exposed to tail suspension increase our knowledge of the hepatic changes in rodents and humans in space. Furthermore, new results regarding *E. gracilis* and its impact on life support systems are published. Finally, the importance of bioactive lipids for the known dysfunctional immune homeostasis and inflammatory damage were reviewed.

I am convinced that space research using the ISS, as well as devices for s-µ*g* in combination with OMICS technologies, will be useful in protecting human health in space during deep space exploration missions and will also be applicable to translational regenerative medicine on Earth.

## Figures and Tables

**Table 1:** Contributions to the Special Issue “Effects of Weightlessness on Molecular Changes in Cellular Organisms, Animals and Plants”.

Author	Title	Topics and Results	Type	Reference
Jokšienè J. et al.	Effects of high glucose on human endothelial cells exposed to simulated microgravity	Human EA.hy926 endothelial cells exposed to a 3D clinostat and to NG or HG for 14 d. Glucose concentrations had a weaker effect than clinorotation. µ*g* downregulated the ECM gene and protein expression and had a stronger influence on glucose metabolism than HG. HG caused more pronounced changes in 3D cultures than in 2D cultures, including an increase in the size and number of spheroids, upregulation of NOX4 and NF-kB and CASP3, and downregulation of fibronectin and transglutaminase-2.	Research article	[14]
Tran V. et al.	Oligodendrocyte progenitors display enhanced proliferation and autophagy after space flight	Behavior of oligodendrocyte progenitors (OLPs) after spaceflight using time-lapse microscopy. Space-flown OLPs presented a significant increase in autophagic cell death. Secretomics: heat shock protein-90 beta was the fifth most enriched (6.8 times) and the secreted protein acidic and rich in cysteine “SPARC” was the seventh most enriched (5.2 times) relative to ground control cells.	Research article	[15]
Veliz A. et al.	Transcriptomic effects on the mouse heart following 30 days on the international space station	Transcriptomic changes (RNA sequencing): hearts of female C57BL/6J mice flown on the ISS for 30 days. A total of 1147 transcripts were significantly regulated after spaceflight: MAPK, PI3K-Akt and GPCR signaling pathways were predicted to be activated. Transcripts related to cytoskeleton breakdown and organization were upregulated. No significant change in ECM components or oxidative stress pathway-associated transcripts. Absence of cellular senescence and upregulation of cell cycle genes. Cellular maintenance and survival were most affected, suggesting that cardiovascular transcriptome initiates an adaptive response to long-term spaceflight.	Research article	[16]
Steinwerth P. et al.	Structural and molecular changes in human chondrocytes exposed to the rotating wall vessel bioreactor	RWV bioreactor to culture primary human chondrocytes and cells from the immortalized line C28/I2 for up to 14 d. Spheroid formation starting on day 3. After 14 d, size of C28/I2 spheroids: 5 mm, primary chondrocyte spheroids 3 mm, characteristic cartilage morphology with collagen-type 1, -type 2 and -type 10 positivity. Primary chondrocytes: significant changes in *IL6*, *ACTB*, *TUBB*, *VIM*, *COL1A1*, *COL10A1*, *MMP1*, *MMP3*, *MMP13*, *ITGB1*, *LAMA1*, *RUNX3*, *SOX9* and *CASP3* gene expression. C28/I2 cells: No changes in gene expression. The RWV bioreactor is suitable for engineering dense 3D cartilage-like tissue without any scaffolds.	Research Article	[17]
Carpo N. et al.	Space flight enhances stress pathways in human neural stem cells	Space (SPC)-flown neural stem cells (NSCs) cultured on the ISS for 39.3 d and then returned to Earth. Postflight: Most of these cells survived the spaceflight and self-renewed. Some NSCs showed enhanced stress responses and autophagy-like behavior. The secretome from SPC-flown NSCs contained molecules inducing these responses. Incubation of NSC resulted in a four-fold increase in stress responses. Proteomic analysis of the secretome: elevated SPARC protein.	Research Article	[18]
Krüger J. et al.	Changes in gravitaxis and gene-expression in an Euglena gracilis culture over time	Microarray analysis of 5-day-old *E. gracilis* cells with positive gravitaxis, 6-day-old cells without gravitactic orientation (controls) and older cells (9- and 11-day-old with negative gravitaxis). A total of 2779 transcripts were differentially expressed. Positive gravitactic cells (5-day-old culture) showed only minor differences in gene expression compared to controls. mRNAs changes were found in older cells. Genes coding for adenylyl cyclases, photosynthesis, and metabolic enzymes were differentially expressed. *E. gracilis* is important for biotechnology and life-support systems.	Research Article	[19]
Ru M. et al.	Integration of proteomic and metabolomic data reveals the lipid metabolism disorder in the liver of rats exposed to simulated microgravity	S-µ*g* rat model: standard rodent tail suspension model; focus on liver tissue. Proteomics and metabolomics: 63 proteins and 150 metabolites between the s-µ*g* and controls. S-µ*g*: dysregulation of major metabolic pathways (biosynthesis of unsaturated fatty acids, linoleic acid metabolism, steroid hormone biosynthesis and butanoate metabolism).	Research Article	[20]
Barkia B. et al.	The formation of stable lung tumor spheroids during random positioning involves increased estrogen sensitivity	Cancer research under s-µ*g* (RPM): Spheroid formation to study metastasis in vitro. Two cell types of lung cancer (NCI-H1703 squamous cell carcinoma cells and Calu-3 adenocarcinoma cells). NCI-H1703: spheroids after 3 d, Calu-3: 2D cell layer after 3 d. Two-dimensional growing Calu-3 cells have less mucin-1, further downregulating their expression on the RPM. Calu-3 cells form unstable spheroids due to an imbalanced ratio of adhesion proteins (β1-integrin and E-cadherin) and mucin-1. RPM-exposed Calu-3 cells showed a strongly upregulated ESR1. 17β-estradiol or phenol red induced more stable Calu-3 spheroids.	Research Article	[21]
Kahlert S. et al.	Dynamic mechanical load as a trigger for growth and proliferation in porcine epithelial cells	Porcine epithelial cells (IPEC-1 and IPEC-J2) exposed to dynamic cultivation systems to mimic the load changes and the resulting mechanical forces. Changes in morphology in IPEC-1 but not in IPEC-J2. IPEC-1 changes in the distribution and structure of F-actin cytoskeleton. Apical brush border and tight junctions were unaffected; decrease in transepithelial resistance inIPEC-1 and partially affected IPEC-J2; increase in Ki67. Doubling of cellular mitochondrial respiration. No signs of senescence.	Research Article	[22]
Fava M. et al.	Cellular and molecular effects of microgravity on the immune system: A focus on bioactive lipids	Focus on the effects of real and simulated µ*g* on the metabolism and signaling of bioactive lipids in the context of immune and inflammatory homeostasis.	Review	[23]

## Data Availability

Not applicable.

## Abbreviations

The following abbreviations are used in this manuscript:

*ACTB*	Actin Beta gene
2D	Two-dimensional
3D	Three-dimensional
*CASP3*	Caspase-3 gene
*COL1A1*	alpha-1 chain of type I collagen gene
*COL10A1*	alpha-1 chain of type I0 collagen gene
D	Days
*E.*	Euglena
ECM	Extracellular matrix
ESA	European Space Agency
ESR1	Estrogen receptor 1
GBFs	Ground-based facilities
GPCR	G protein-coupled receptors
HG	High-glucose medium
HSP-90	Heat shock protein 90
*IL6*	Interleukin-6 gene
ISS	International Space Station
*ITGB1*	Integrin beta 1 gene
*LAMA1*	Laminin subunit alpha-1 gene
MAPK	Mitogen-activated protein kinase
µ*g*	Microgravity
min	Minutes
*MMP1*	Matrix Metallopeptidase 1 gene
*MMP3*	Matrix Metallopeptidase 3 gene
*MMP13*	Matrix Metallopeptidase 13 gene
NASA	National Aeronautics and Space Administration
NG	Normal glucose medium
NSC	Neural stem cells
OLPs	Oligodendrocyte progenitors
PI3K-Akt	Phosphoinositide 3-kinase-protein kinase B
PPAR	Peroxisome proliferator-activated receptor
RPM	Random Positioning Machine
*RUNX3*	Runt-related transcription factor 3 gene
RWV	Rotating Wall Vessel
SI	Special Issue
s-µ*g*	Simulated microgravity
SOX9	SRY-Box Transcription Factor 9
SPARC	Secreted protein acidic and rich in cysteine (osteonectin)
*TUBB*	Tubulin Beta gene
*VIM*	Vimentin gene
